# Bioregulatory systems medicine: an innovative approach to integrating the science of molecular networks, inflammation, and systems biology with the patient's autoregulatory capacity?

**DOI:** 10.3389/fphys.2015.00225

**Published:** 2015-08-19

**Authors:** Alyssa W. Goldman, Yvonne Burmeister, Konstantin Cesnulevicius, Martha Herbert, Mary Kane, David Lescheid, Timothy McCaffrey, Myron Schultz, Bernd Seilheimer, Alta Smit, Georges St. Laurent, Brian Berman

**Affiliations:** ^1^Concept Systems, Inc.Ithaca, NY, USA; ^2^Department of Sociology, Cornell UniversityIthaca, NY, USA; ^3^Biologische Heilmittel Heel GmbHBaden-Baden, Germany; ^4^Transcend Research Laboratory, Massachusetts General HospitalBoston, MA, USA; ^5^International Academy of Bioregulatory MedicineBaden-Baden, Germany; ^6^Division of Genomic Medicine, George Washington University Medical CenterWashington, DC, USA; ^7^St. Laurent InstituteVancouver, WA, USA; ^8^Center for Integrative Medicine, University of Maryland School of MedicineBaltimore, MD, USA

**Keywords:** bioregulatory systems medicine, systems biology, autoregulation, inflammation, systems medicine, genomics

## Abstract

Bioregulatory systems medicine (BrSM) is a paradigm that aims to advance current medical practices. The basic scientific and clinical tenets of this approach embrace an interconnected picture of human health, supported largely by recent advances in systems biology and genomics, and focus on the implications of multi-scale interconnectivity for improving therapeutic approaches to disease. This article introduces the formal incorporation of these scientific and clinical elements into a cohesive theoretical model of the BrSM approach. The authors review this integrated body of knowledge and discuss how the emergent conceptual model offers the medical field a new avenue for extending the armamentarium of current treatment and healthcare, with the ultimate goal of improving population health.

## Introduction

For over a decade, discoveries in systems biology have catalyzed new waves of thinking in medicine. Systems and network theory, coupled with advances in technologies analyzing vast datasets, are propagating novel perspectives of human health, disease, and patient treatment. In this paper, we define a systems approach as a method for describing the human body as a complex biological network of interconnected components (molecules, cells, tissues, organs). Since the turn of the century, scientific communities around the globe have been driven by the revolutionary insights garnered from the Human Genome Project. Many recently established initiatives aim to translate these insights in a way that is practically relevant to medical treatment (P4 Medicine Institute, 2012; Cesario et al., 2014; CASYM^1^). A key area of ongoing research relates to expanding empirically-based clinical knowledge that supports the applicability of systems medicine concepts. A unidirectional, discovery-oriented approach undoubtedly results in innovative diagnostic and therapeutic solutions. We suggest that a more bidirectional approach that connects the dots between scientific discoveries and clinical application may also reveal some important medical innovations that are highly relevant to a broad range of healthcare practitioners.

In this paper, we introduce a medical paradigm that further develops insight from systems biology by merging its key scientific principles with relevant empirical evidence into a treatment model. We call this approach “bioregulatory systems medicine” (BrSM). BrSM is rooted in the idea that a more robust and effective solution for disease complexity should optimize an individual's homeostatic systems and their interactions across all levels of biological organization. From the detailed picture of protein homeostasis at the molecular level, for example, to temperature and blood pressure regulation at the broader, whole-organism level, the interconnected web of these systems and their impact on individual health status constitutes a cardinal focus of this therapeutic approach.

Like other paradigms grounded in systems biology, BrSM emerges in part as a response to the limitations of the reductionist perspective that is central in the current healthcare model (Tillmann et al., 2015). The reductionist perspective tends to view the human organism as a compilation of targets for individual intervention and symptom alleviation. Clinicians typically specialize in particular fields focused on single systems or tissues of the body, and concentrate largely on symptom expression in evaluating and treating disease (Stange, 2009).

Since the widespread adoption of the current healthcare model in the 1960s, medical costs have escalated as much as 15 times (Gaygisiz, 2010), and rates of chronic disease are projected to increase more than 50% by 2023 (Bodenheimer et al., 2009). Nonetheless, medical care is currently estimated to account for only 10% of health outcomes, while as much as 80% are influenced by environmental and lifestyle factors (McGinnis et al., 2002). It behooves the field to consider how medical care can effectively address the impact of these environmental and lifestyle factors that have such profound influences on health (Miller and Jones, 2014). We propose that the medical and financial burdens associated with the current healthcare model can ultimately be tied to neglecting a basic tenet of systems biology in clinical care: relatively simple network perturbations can have large and unintended consequences. Indeed, the prevailing linear mode of intervention can be linked to increasing rates of iatrogenesis, unnecessary diagnostics, and multiple practitioner consultations, which collectively contribute to escalating healthcare costs and inefficiencies in patient treatment (Ahn et al., 2006; James, 2013). These inefficiencies, in turn, contribute to the growth in disease incidence that burdens society today, which is arguably more complex than what can be explained and resolved by the reductionist approach.

The significant contributions of the dominant healthcare approach cannot be diminished. Emergency and acute care, vaccines, surgery, and preventative medicine are among the major healthcare advances of the past century. Nonetheless, the specialized context within which many clinicians treat patients today, combined with limited incorporation of biological complexity as part of treatment, has curtailed the capacity to resolve many chronic and lifestyle-related diseases that manifest at the systems level. The complexity of the human organism cannot be reduced to a parts list of molecules, which yields little functional understanding of regulatory networks (Oltvai and Barabási, 2002). Instead, genomics and computational network modeling must be used to better understand and treat diseases from a more integrated, higher-order perspective.

**Figure 1:**
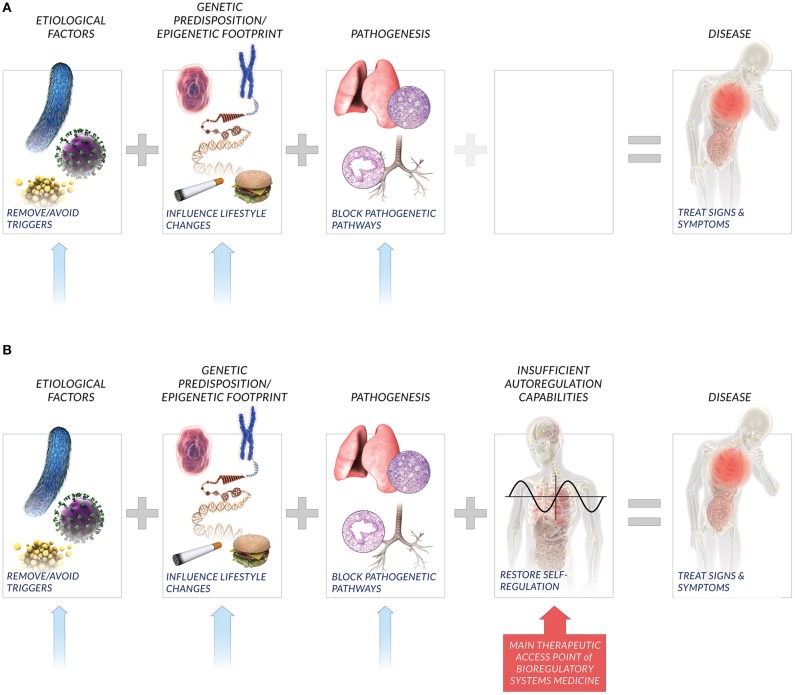
**New perspective on factors affecting disease**. Current medical paradigms **(A)** typically consider etiological factors, genetic predisposition, and molecular pathways recruited in pathogenesis as key causative agents that lead to disease. Bioregulatory systems medicine also considers the compromised or insufficient patient autoregulatory capacity to restore homeostasis **(B)** as a key factor that influences individual disease incidence and manifestation. Relatedly, bioregulatory systems medicine includes the interactions among multi-scale homeostatic systems that comprise the global autoregulatory network as the primary therapeutic access point in treating signs, symptoms, and underlying causes of individual disease. While lifestyle changes, removal of triggers, and inhibition of pathogenetic pathways are also potential solutions, bioregulatory systems medicine emphasizes the restoration of individual autoregulation capacity as the potent therapeutic approach.

At the heart of BrSM is an appreciation of the patient autoregulatory capacity in light of lessons from systems biology. Whereas human biology exists as nested levels of physiological networks, i.e., molecular, cellular, tissue, organ, system, etc., the autoregulatory capacity includes both the specific networks within this hierarchy as well as the interactions between them. BrSM embraces this interconnectivity among networks as the global autoregulatory network, and posits that the state of an individual's autoregulatory network is a key determinant and indicator of patient health. The patient autoregulatory network is also a key therapeutic access point in BrSM (Figure 1).

Until now, the concept of BrSM has existed as a loose set of scientific, clinical, and empirical evidence that support the general idea of autoregulation and its role in health and disease. Against the backdrop of systems biology, we aim to begin answering the questions: What are the details of these concepts that are pertinent to a cohesive approach that can improve clinical outcomes? How are these details interrelated, and what is the significance of this interrelatedness for advancing therapeutic practice? Consequently, a team of scientists and clinicians set out to systematically bring together those elements most critical to BrSM, and to engage in an initiative to conceptualize an integrated, cohesive model of this approach. An emphasis on multiple disciplines not only ensured that cutting-edge research from various fields was included in formalizing the paradigm, but also that the model was more likely to resonate with and demonstrate applicability to a broad community of scientists and clinicians.

The purpose of this paper is to present the BrSM model that resulted from this initiative. This model lays important groundwork for scientists and clinicians to begin the research and data collection necessary to fully realize the potential advantages of this approach. For a medical community that has been educated, practicing, and thinking within a largely reductionist framework for some time, the shift toward incorporating a systems biology view of the human organism can be challenging. We envision BrSM and the underlying principles in this model as a strategic guide for connecting and applying emerging research in a way that will lead clinicians toward realizing improvement in patient outcomes.

## Materials and methods

**Figure 2:**
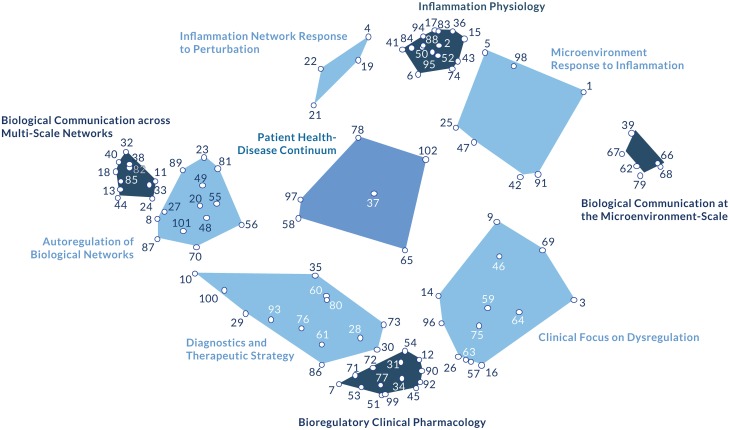
**The bioregulatory systems medicine model**. This figure depicts the 10 constructs that emerged from the concept mapping process, reflecting expert consensus on the key elements of the bioregulatory systems medicine approach. Underlying the constructs (clusters) are the 102 statements that comprise the basic scientific and clinical elements of the approach. The labels assigned to each cluster reflect the shared theme of its specific statements and in relation to the contents of every other cluster (Supplementary Table 1). From a more empirical perspective, the 10 model clusters can also be considered within four thematic groupings. The autoregulation clusters (*Biological Communication across Multi-Scale Networks, Biological Communication at the Microenvironment-Scale*, and *Inflammation Physiology*) describe the physiological autoregulation of biological networks. The dysregulation clusters (*Inflammatory Network Response to Perturbation* and *Microenvironment Response to Inflammation*) describe the biological networks' response to perturbation. The central position of *Patient Health-Disease Continuum* conveys its influence on both the autoregulation and dysregulation clusters. This concept can be interpreted as a correlation between dysregulation and autoregulation, representing the resultant disease state of a patient along the health-disease continuum. The adjacent cluster, *Autoregulation of Biological Networks*, reinforces the concept of autoregulation as the common, systems biology-based denominator underlying the model. The remaining clusters are therapy-related. *Diagnostics and Therapeutic Strategy* links a patient's autoregulatory status with clinical decision-making. *Clinical Focus on Dysregulation* describes local aspects of dysregulation, suggesting that certain molecular networks can be targeted using bioregulatory therapeutics depending on the nature of their dysregulation. *Bioregulatory Clinical Pharmacology* connects clinical decision-making and intervention tactics.

Fundamental to this initiative was first selecting the appropriate range of participant expertise and professional experience to contribute to the model development, and then engaging these viewpoints in a group conceptualization process that would systematically integrate the key elements into a cohesive model. The following steps were taken to achieve these goals:

1. Initiative leaders identified and approached participants that collectively possessed considerable breadth and depth of relevant expertise. Scientific experts included those in the fields of immunology, genomics, molecular biology, neuroscience, and systems biology. Clinicians specializing in various medical areas were also involved, including family and community medicine, chronic diseases, aging, cardiology, pediatrics, and neurology.
2. Group concept mapping was selected as the most appropriate method for the model development process given its prior use in similar projects (Baldwin et al., 2004; Kane and Trochim, 2007; Kagan et al., 2009). The method is capable of systematically integrating particularly complex ideas, perspectives, and their relational properties, such as those used to create this model. The group-authored visual outputs are based entirely on the combination of individual participant perspectives, and provide fertile ground for generating group consensus on the interpretation and meaning of the results.
3. Participants generated model content (statements) in response to a single guiding sentence completion: “A specific idea or element that is fundamental to defining and explaining a model of BrSM is…” Content was generated through an iterative process of abstraction from focused literature review and discussion and refinement with expert participants.
4. Participants individually sorted the resultant 102 statements into piles based on their own understanding of their relatedness and using a dedicated project website. Readers are referred to Kane and Trochim (2007) for a detailed description of the sorting process.
5. Analysis of participant sorting arrangements included aggregation of individual binary sort matrices (a “1” was placed in a cell if a participant sorted the statements in the corresponding row and column into the same pile; a “0” was placed in a cell if the participant did not sort the corresponding statements together). The 29 participant sort matrices were then summed, creating a total similarity matrix. The total similarity matrix was then subjected to multi-dimensional scaling, producing a two-dimensional visual representation (point map) of the 102 statements. Proximity among statements indicates their relative similarity, such that the closer two statements appear on the point map, the more similar or related they are thought to be by the group as a whole, and vice versa.
6. Hierarchical clustering was applied to the point map to group statements in shared territories of the map into non-overlapping clusters based on the Euclidian distances between them. The smaller set of resulting clusters allows participants to consider the model through the lens of higher-order themes that capture the specific details of the underlying content. A 10 cluster solution was determined by initiative leaders to be the most parsimonious and meaningfully interpretable representation of the model content.
7. Finally, initiative leaders and participants reviewed the cluster map as part of a multi-day meeting, and labeled each cluster in a way that articulated the commonality among its constitutive statements, and in a way that conveyed the constructs' relevance in the context of the model as a whole (Figure 2) (Supplementary Table 1).

## Results: Interpretation of the bioregulatory systems medicine model

From the group concept mapping process, a two-dimensional point map was generated to visualize the emergent group consensus. The cluster map represents the 102 statements as they are grouped into 10 higher-order themes based on their arrangement in the point map (Figure 2). Cluster analysis revealed four anchor themes (*Inflammation Physiology, Biological Communication at the Microenvironment-Scale, Biological Communication across Multi-Scale Networks, Bioregulatory Clinical Pharmacology*) and six intermediary themes (*Inflammatory Network Response to Perturbation, Microenvironment Response to Inflammation, Diagnostics and Therapeutic Strategy, Clinical Focus on Dysregulation, Autoregulation of Biological Networks, Patient Health-Disease Continuum)*. From these conceptual patterns, two axes emerged. The horizontal Biological Information axis suggests how clinicians evaluate characteristics of the disease, serving as a guide for clinical decision-making, while the vertical Resolution Processes axis considers mechanisms of intervention.

As we move to our interpretation, detailing the conceptual basis of the model axes and clusters, we remind readers that the unique scope and content of each cluster exists as an emergent product of the expert participants' perspectives. The cluster arrangements that underpin this interpretation could each plausibly constitute their own paper, as each include their own unique set of scientifically grounded statements that could be explored in depth. Because this paper is an introduction to the BrSM concept, we center our discussion on reviewing participants' perceptions of the content interrelatedness, and the emergent themes that capture how the key components of the paradigm formulate an organized conceptualization of treatment.

The collective spatial properties of the model also convey emergent properties that reflect participants' integrated understanding of the approach's medical and clinical components. At the broadest level, we can examine conceptual patterns that reveal how the content is distributed across the two-dimensional model representation, and consider the meaning of this distribution in terms of its practical and theoretical implications.

The content closest to the *Biological Communication at the Microenvironment-Scale* cluster relates most strongly to communication and signaling at the cellular level, particularly as it occurs within and by way of the extracellular matrix. The content located closest to the *Biological Communication across Multi-Scale Networks* cluster resonates with a systems-level understanding of how information flows between molecular networks/organ systems at the whole organism level. Collectively, this conceptual through-line contains elements related to the role of biological information at both relatively “micro” and “macro” levels. We label this dimension (or axis) as Biological Information, across which informational content is present at varying levels of specificity depending on its position along the dimension and in relation to other information-related elements of the model.

Perpendicular to Biological Information, experts distinguished among relatively internal and external resolution mechanisms. Closest to the *Inflammation Physiology* cluster, the model content relates strongly to the human organism's natural ability to reach resolution in the face of perturbation, particularly as it relates to inflammation process mechanisms. At the opposite end of this dimension, the *Bioregulatory Clinical Pharmacology* cluster describes the use and application of therapeutics in the clinical context in order to reach resolution. As all content along this axis relates in some manner to participants' conceptualization of resolution, we label this axis of the map as Resolution Processes. We recognize that in some cases resolution pertains to the organism's innate capacity to reach resolution, and in other cases to the use of external interventions as part of treatment.

Even more specifically, those clusters most centrally aligned on either end of each dimension serve as conceptual anchors that ground the BrSM model in its key clinical focuses. The *Inflammation Physiology* and *Bioregulatory Clinical Pharmacology* clusters convey the “how” of physiological coherence and restoration, leading clinicians to explore questions about intervention such as: *How* do the inflammatory processes function to influence autoregulation, and what are the physiological factors involved? *How* should bioregulating medications be designed and applied to effectively restore autoregulation?

Likewise, the *Biological Communication at the Microenvironment-Scale* and *Biological Communication across Multi-Scale Networks* clusters specify those elements necessary to understand the range of biological signaling and communication pathways that underlie autoregulation. This content prompts the clinician to explore questions of: *What* is taking place at the cellular or “micro” level of the human organism that influences regulatory capability? *What* is taking place at the network, or “macro” level to influence regulation across systems? At the “micro” level, emphasis is placed on the role of the extracellular matrix in pathological conditions, particularly with regard to the accumulation of toxins, disease progression, and transcription patterns. At the “macro” level, information and signaling across molecular networks direct regulatory action among organ systems, such that the large-scale complexity of the cellular-level interactions can be understood as an integrated, interconnected picture of human health.

The emergence of these clusters as conceptual anchors is also validated methodologically. Structurally (spatially), these four clusters are more densely populated with statements than the other clusters of the map, indicating that participants perceived a higher degree of conceptual similarity among the set of items in each of these four clusters relative to the other clusters. The density of these clusters implies a high degree of consensus from experts, suggesting that participants collectively understood a greater degree of clarity and distinctiveness in the meaning of these sets of items relative to the other clusters. Functionally, these clusters demonstrate the highest degree of internal relatedness, indicating that participants understood the statements in each of these four clusters as more strongly related to one another and less related to the statements in the other clusters of the model. These clusters are also the functional anchors of the map in the sense that they function as the cohesive, agreed-upon, foundational classes of information from which the conceptual role of the other six clusters can be considered.

Experts considered the other six clusters as conceptual bridges that articulate relationships among the anchors that they reside between. Structurally, these clusters occupy a comparatively larger area of the map and are overall less densely populated with statements than the anchors. Their relatively expansive area suggests that participants perceived considerable similarity among the set of items in these clusters and the set of items in their respective adjacent anchors. Their value in the approach is optimally derived from their ability to build coherence among the anchor constructs, and logically bridge the core elements in a way that can be practically applied in the clinical context. Thus, we refer to these six clusters as the intermediary clusters.

*Microenvironment Response to Inflammation*, for example, brings together the physiology of inflammation with “micro” or local level information regulation to describe the environment in which inflammation initiation and resolution take place. *Inflammatory Network Response to Perturbation* articulates the mechanisms of inflammation with a more thorough understanding of the systemic and informational components of this physiological process. *Diagnostics and Therapeutic Strategy* conveys the practical use of “macro” or global network level information in the design and application of medication with bioregulatory properties. This cluster emphasizes the use of diagnostics, such that autoregulatory networks can be appropriately assessed and, in turn, interpreted in a way that will effectively guide treatment. The content in this area also highlights the use of diagnostics for furthering our knowledge of disease evolution and thereby enhancing strategic therapeutic decision-making. *Clinical Focus on Dysregulation* identifies specific conditions and pathologies for which BrSM is well suited, although additional content, particularly regarding toxicity, may be helpful in fully realizing the relationship between the extracellular matrix and the clinical context.

*Patient Health-Disease Continuum* occupies a unique position in the center of the map, where one can envision the intersection of the Resolution Processes and Biological Information axes. This cluster emerges as the “hub” that personalizes the theoretical foundation of the model, emphasizing the individual, patient-centric basis of BrSM. As the structural core, this construct includes clinician considerations for optimizing resolution, as well as critical biological information for the clinician to consider in improving patient condition. Symptoms, disease progression, autoregulatory abilities, and inflammation are informative expressions of an individual's health status that can be used to personalize treatment.

These thematic patterns reveal principles of the BrSM model that are rooted in experts' understanding of the elements' interrelatedness, beyond the statement and cluster content alone. To summarize the model results at the theoretical level, the bioregulatory systems approach is driven by the goal of stimulating resolution processes through the communication and information pathways of the human organism. The model conveys that a clinically integrated picture of biological information, when utilized to restore coherence following perturbation, constitute the two fundamental concepts for approaching patient disease using BrSM.

We now move to explore in greater depth the key principles of BrSM that connect the model results with a clinically relevant understanding of this approach.

## Key principles of the BrSM approach

### A network structure of health and disease: The influence of molecular network information flow on autoregulatory capacity

**Figure 3:**
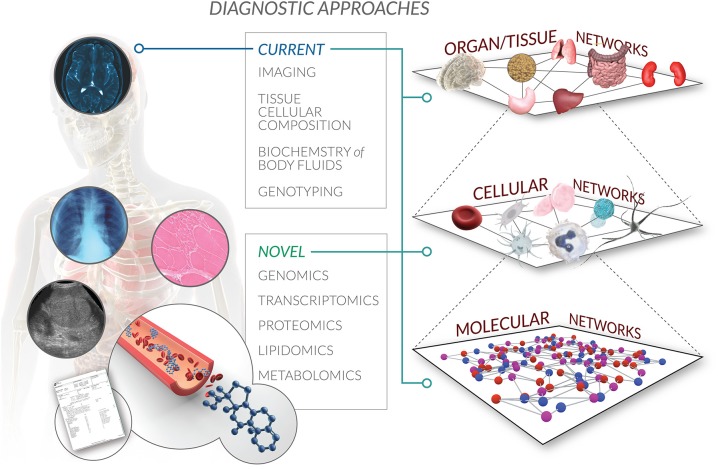
**Multi-scale autoregulatory networks in the BrSM model**. Bioregulatory systems medicine encompasses a systems biology perspective of interactions within and across multiple levels of biological organization. From the molecular to cellular to organ to whole organism network, the BrSM model acknowledges that human health and disease are driven by the regulatory information flow that propagates throughout this global autoregulatory network. Current diagnostic approaches are limited by capturing only a static snapshot of some of this information recorded as medical records. Novel diagnostic approaches will not only confirm and provide higher resolution of existing snapshots of clinical information, but will also expand the scope of medical records by adding (surrogate) biomarkers of autoregulation that will correlate all captured information in one spatiotemporal model specific to the patient.

It comes with little surprise that participants recognized the complexity intrinsic to biological systems as a core feature of the BrSM model. Given that systems biology serves as a primary scientific backdrop that fuels this approach, a holistic understanding of the human body as a multi-scale, multi-level regulatory network percolates the entire concept map (Hunter et al., 2002; PacificBiosciences, 2011; Castiglione et al., 2014) (Figure 3). The complexity of a systems approach challenges common reductionist thinking, and paves the way for medicine that works *with* rather than *against* the inherent interconnectivity of biological organization.

Participants conceptualized health and disease in the context of this interconnectivity. A healthy system is one that self-regulates in the face of network perturbation (Buchman, 2002). We refer to this self-regulation as autoregulation. Model participants embraced autoregulatory capacity as a defining mechanism of human health. The concept of robustness is used in tandem with autoregulation to characterize the functional characteristics of a healthy system. Whereas biological networks are inherently dynamic and unstable, a robust system is one that is able to adapt to and cope with this instability as it is received from the environment (Kitano, 2004, 2007a,b; Kitano et al., 2004). Some readers may liken the concept of robustness to homeostasis; however, participants distinguished that in the context of BrSM, homeostasis is a property that *maintains* the state of a system, whereas robustness assumes a more dynamic, active network state, and refers to sustained system functionality, even in the face of stresses or perturbations. Individual autoregulatory abilities support network robustness. Metabolic, gene and protein networks interconnect to create a global biochemical network, while the feedback loops across these networks constitute the foundation of the global autoregulatory network of the human organism (Droujinine and Perrimon, 2013).

Disease occurs when an individual's autoregulatory abilities are compromised. We encounter this scenario when accumulated stresses overpower the autoregulatory abilities, thereby impinging tissue robustness. These persistent perturbations can manifest as disease over time (Schadt, 2009; Furlong, 2013; Baffy and Loscalzo, 2014; Vuillon and Lesieur, 2015). As humans, we regularly encounter genetic, epigenetic, and environmental perturbations apart from others that challenge the robustness of our biological networks. This continuous challenge may negatively impact autoregulatory ability, and lead to a “rewiring” of these networks to new adaptation and compensation states. Over time, these negative impacts may gradually progress to eventually result in disease.

Participants also linked this conceptualization of disease with biological networks, considering the consequences of this interconnectivity for disease progression. These consequences are evident in the case of an inflammatory response, where chronic inflammation coincides with structural changes in a tissue and in remodeling the microenvironment (Nathan, 2002; Nathan and Ding, 2010). Given that many tissues and organs are connected via networks of functional interdependencies, stresses or perturbations that compromise autoregulatory abilities can cause a ripple-like effect throughout networks of interconnected tissues. This propagation facilitates disease progression by proliferating distorted information flow.

The concept of modularity is used to propose how pathophysiological events tend to organize and the impact of this organization on health maintenance. Modules are self-organized units of individual components that are grouped according to a certain set of rules (e.g., a common function), and that allow networks to optimize their dynamics and adapt to disturbances (Newman, 2006; Loscalzo et al., 2007; Rollié et al., 2012). Modularity helps networks to contain perturbations, support autoregulation, and minimize the effects of disease on the system (Kitano, 2004).

As many diseases are interconnected by shared pathophysiological events, recent research has identified a common network that is perturbed in the majority of chronic diseases. We call this network a “common disease-state signature” (Suthram et al., 2010; Janjić and Pržulj, 2012). Many diseases share common functional modules, suggesting that treatment courses and medications may be more effective by targeting these biological networks rather than the historically more common approach of targeting single molecules. This overlap across biological networks is a particularly advantageous framework for understanding the aging process and age-related diseases in novel ways. Multitarget drugs that target hubs, bridges, or other areas of network overlap may therefore be more effective and lead to fewer side effects than the more common single-target, “magic bullet” drug design (Simkó et al., 2009). From the perspective of BrSM, optimal therapeutic access points may be discovered by utilizing these pathological threads between seemingly unrelated diseases. This pathophysiological connectivity may be a pathway to supporting a future of a network pharmacology (Erler and Linding, 2010; Barabási et al., 2011; Li et al., 2011), where networks themselves are the focus of medication design and therapies.

Experts also acknowledged that sustained perturbation in information flow (e.g., blocks to autoregulation) precipitates the inability of regulatory networks to maintain functionality. Indeed, the transmission of biological information maintains a cardinal role in supporting and regulating the dynamic equilibrium found in robust networks.

What is the nature of biological information most relevant to BrSM? Participants viewed information theory and thermodynamics as fundamental for understanding the medicinal relevance of a biological system. Beyond these areas that are already well integrated into systems biology, participants considered two broad categories of biological information as supporting system robustness. The first category includes sequence information that is responsible for encoding molecular machineries. This type of information is often referred to as the “parts list” of sequence information that reductionist medicine techniques have decoded.

Additionally, regulatory network information is thought to be responsible for orchestrating the particular behaviors of molecular machineries, and is thought to be transmitted by non-coding RNA across levels of biological structures (molecules to cells, cells to tissues, etc.). A theory of genomic dark matter also surfaced on the concept map, positing that a cell's dynamic response to inputs from the microenvironment is governed by non-coding, RNA-regulated molecular machineries (St. Laurent et al., 2012). Participants believed that these molecular machineries support the notion that a cell's interaction with its immediate environment is in fact a coherent pattern, and is not a disordered or otherwise chaotic flow of molecules as some might assume. This coherence is thought to be sustained in part by a computational matrix that directs action within and across molecular networks, and exists as a result of low affinity RNA and protein interactions. Recent research lends further support to the role of non-coding RNAs in health and disease. For example, non-coding RNAs may mediate stress response pathways of some diseases such as Alzheimer's (St. Laurent et al., 2009), and are secreted by immune cells, stem cells, adipocytes, and blood cells (Chen et al., 2012). From a diagnostic perspective, the presence of non-coding RNAs in serum and other bodily fluids may suggest their potential as clinical biomarkers (Iorio and Croce, 2012).

Both macro and micro scales of information regulation hold prominent places in this model. At the most expansive level, the interconnectivity of molecular networks creates a global biochemical network containing numerous function-specific networks and feedback loops. In BrSM, health-disease status is governed by the particular integration of these networks as the global autoregulatory network, and the orchestration of their responsiveness to environmental stimuli.

Whereas systems level information regulation is a critical concept, participants also considered the cellular scale of information regulation. The role of the microenvironment as a critical supporter of healthy cells (Buttle, 2007) and as a conduit of biological information in tissues (Nathan and Ding, 2010) was regarded as fundamental. A healthy microenvironment encompasses the biochemical and biophysical signals that a cell receives from the extracellular matrix, neighboring cells, and the immune system, and is necessary for a cell and tissue to maintain their function and autoregulatory ability (cell turnover) (Pellettieri and Sánchez Alvarado, 2007; Duarte et al., 2015; Fu et al., 2015; Mesa et al., 2015). Regulation at this more micro level takes place within the tissue via the extracellular matrix, intracellular cytoskeleton, and nuclear matrix, all of which are interconnected by commonly utilized molecules. The extracellular matrix can be considered an “information highway,” where biochemical, physical, and neural signals are processed and subsequently affect network robustness. In addition to robustness, signaling within the microenvironment supports other processes such as immunological synapse formation in the immune system (Springer and Dustin, 2012; Dustin, 2014), and others related to inflammation (Loscalzo et al., 2007; Dustin, 2012). We explore the role of the extracellular matrix in inflammatory processes in more depth in the subsequent section.

### Inflammation as a central regulatory mechanism for maintaining tissue homeostasis

From a pathophysiological perspective, inflammation is a common feature of many disorders and is strongly associated with chronic and age-related diseases that continue to escalate in incidence under the current healthcare model. Inflammation is traditionally viewed as something to be reduced or suppressed (Widgerow, 2012; Women's International Pharmacy, 2012). BrSM puts forth a far more comprehensive and dynamic view of inflammation, beyond simply a static symptom that needs to be eliminated. In fact, BrSM embraces physiological inflammation as an extension of the autoregulatory capacity of the body, capable of restoring a healthy tissue's functional state (Sansonetti, 2011; Chovatiya and Medzhitov, 2014; Kotas and Medzhitov, 2015; Serhan et al., 2015). The model aids us in more precisely distinguishing among adequate, resolving, and excessive or insufficient inflammatory responses, and in guiding clinicians toward recognizing inflammation as a potential tool in patient treatment selection and a vital part of homeostasis, rather than as the ubiquitous enemy.

Inflammatory mechanisms are switched on by various exogenous and endogenous stressors that aim to eliminate initial stressors and adjust to a changed environment by establishing new homeostatic set-points (Miyake and Kaisho, 2014; Kotas and Medzhitov, 2015). Inflammation can be considered a central physiological mechanism that supports the body's ability to resolve dysfunctional states in order to regain tissue functionality, and in a way that tissues cannot accomplish themselves. Inflammatory processes can therefore play a cardinal role in both disease progression and regression.

Non-resolving, chronic inflammation coincides with its common pathological clinical association, manifesting in chronic diseases such as atherosclerosis, cancer, asthma, diabetes, rheumatoid arthritis, and others (Nathan and Ding, 2010; Hellmann et al., 2012; Young et al., 2014; Fredman et al., 2015). In understanding the inflammatory mechanisms that lead to these pathologies, BrSM also recognizes the intersection between inflammation and information regulation concepts discussed in the prior sections. We find concepts such as balance and systemic effects as extending into this region of the model, reinforcing the notion of biological interconnectivity as an undercurrent of the model as a whole.

Whereas pro-inflammatory mechanisms, anti-inflammatory, and pro-resolution pathways are all involved concurrently to regulate the duration and severity of an inflammatory response, a persistent imbalance of these mediators can lead to pathology and chronicity (Ariel et al., 2006; Beck et al., 2009; Nathan and Ding, 2010; Valledor et al., 2010; Kotas and Medzhitov, 2015; Serhan et al., 2015). For example, inflammatory mechanisms can influence the production of damage-associated molecular patterns (DAMPs), resulting in positive feedback loops in which the inflammatory response itself provides a persistent stimulus for macrophage and lymphocyte recruitment (Foell et al., 2007). This cyclical, excessive production drives host damage and chronic inflammation.

Not all cases of tissue stress or malfunction result in acute inflammatory responses. Insufficient but persistent stimuli can also provoke low grade inflammatory responses. This so called para-inflammation is maintained at a low level without resolution, and can lead to tissue damage and chronic inflammation (Chovatiya and Medzhitov, 2014; Netzer et al., 2015). The model emphasizes that the inflammatory process itself is not dangerous, as it can be resolved by endogenous molecules and mechanisms; rather, it is the non-resolving, chronic or overwhelming acute inflammatory response that leads to pathology. Whereas acute inflammation is typically seen as a target for prevention, BrSM uses this kind of evidence to support the notion of acute inflammation as a homeostatic mechanism that may be supported in a controlled manner to stimulate resolution. To this end, clinicians may find that improving a patient's self-regulatory abilities is a pathway toward attaining resolution initiation naturally. Nonetheless, caution should be taken in overgeneralizing this phenomenon, which may not present itself in other tissues or organs such as the brain, for example.

We underscore participants' recognition that the current healthcare model has historically paid little attention to the inherent connectivity between local and system levels of physiological responses such as inflammation. In BrSM, this connectivity across levels of biological organization is nothing short of essential in understanding disease and improving treatment.

Beyond these pathological distinctions, participants understood inflammation as practically relevant to treatment in two main ways. The inflammatory system is considered the target for stimulating or optimizing disease resolution. The bidirectional communication using inflammatory pathways between a cell and its microenvironment, functions as a homeostatic control mechanism for many tissues. Complete inflammation resolution requires not only the removal of immune cells, but also the normalization of chemokine gradients and the withdrawal of survival signals. Disordered fibroblast behavior, for example, can contribute to chronic non-resolving inflammation by sustaining inappropriate retention of leukocytes within inflamed tissue (Buckley et al., 2001; Buckley, 2011). It therefore seems only reasonable to target the tissue microenvironment in parallel with the stressor and the infiltrating immune cells when treating chronic inflammation (Serhan et al., 2007; Valledor et al., 2010).

With regard to treatment, it is possible that with the ability to mimic or inhibit extracellular matrix functions, we could provide a novel means to influence and resolve chronic inflammation and reveal promising therapeutic targets. Another potential option in treating chronic inflammatory disease might be to permit the restoration of autoregulatory processes in the extracellular matrix, including physiological inflammation, by removing any toxins, stresses, deficiencies, or other perturbations that are interfering with its structure and function. Potentially, this option might also be applicable to cancerous microenvironments. The link between inflammation and tumor development is well established (Sommer, 2014; Blaylock, 2015). Targeting autoregulatory mechanisms that aim to restore original immunosurveillance and neuroendocrine regulation as part of the comprehensive treatment protocol might increase responsiveness of the patient. For an excellent discussion of evolving approaches to cancer management, see McGranahan and Swanton (2015).

In addition to thinking about the microenvironment as a context for intervention, participants hypothesized about how patterns of inflammation and their effects on the microenvironment can be used to assess the state of a patient's autoregulatory network. Because the status of the autoregulatory network is a major player in resolution, it is logical to seek ways of incorporating it into diagnosis. Inflammatory patterns and their effects on the microenvironment may prove a gateway to this type of assessment. We discuss how participants considered this concept as we move to focus specifically on activating the model's scientific principles in the hands of the clinician.

### Incorporating the health-disease continuum into patient diagnosis

BrSM encapsulates a health-disease continuum, along which a patient can be diagnosed in accordance with phases of disease progression, treated via multiple therapeutic access points, and monitored based on how networks manifesting pathophysiological processes resolve to a state of health. Disease progression is the outcome of both the inflammatory response to network perturbation and the effect of this response on the microenvironment. Patient history, physical examination, and laboratory tests are among the sources of information that clinicians can use to determine an individual patient's state along this continuum. We anticipate that as genomic profiling becomes part of routine testing, this data will allow us to be even more specific about where a patient stands along a scale of health and disease, and will also provide us with information on the status of the autoregulatory network (Figure 3).

A goal of BrSM is to eventually develop biomarkers that predict the health-disease continuum, and that match therapeutic interventions to the specific stage of a patient's disease. Whereas historically we have thought about disease in a relatively linear, gene-centric, and deterministic way, we now realize that this perspective is too limiting and is unable to explain either the dramatic rise in the rates of these diseases over the past 50 years or the relatively minimal penetrance of gene mutations in most chronic diseases (Renz et al., 2011). The modern view realizes that extrinsic factors such as nutrition, the microbiome, and the environment, combined with intrinsic factors such as the gut and respiratory mucosa, must be collectively considered as affecting gene expression, possibly via the processes of epigenetics. The complexity of these chronic conditions demands that scientists and clinicians also be complex thinkers, studying the interaction of these factors in a dynamic temporal and spatial way, and defining and describing diseases on the basis of their intrinsic biology in addition to traditional signs and symptoms (National Research Council, 2011). One solution would be rethinking the way of classifying diseases, adding new elements possessing certain predictive value that would allow the clinician to dynamically monitor patient condition and adjust treatments in a timely manner. From that perspective, the BrSM model proposes incorporating autoregulatory capacity assessment as part of the diagnosis, providing additional information to further individualize the treatment plan.

### Creating a more effective clinical toolbox by choosing the right therapeutic strategy, clinical focus, and bioregulatory intervention

As we explore the remaining areas of the model, concepts such as the inflammatory and microenvironment responses to network perturbation, disease progression, and autoregulation serve as key insights for more fully realizing how BrSM may actually propagate changes in medical treatment. In this section, we discuss how the strategic implementation of BrSM approaches the patient condition in ways that are distinct from the current healthcare approach.

When presented with a case, a clinician using the BrSM approach will initially assess whether the patient exhibits an inflammatory response to network perturbation at the systems level. Inflammatory responses can vary in manifestation from mild to moderate acute, severe acute, acute with transition to chronic, and chronic. The actual nature of an inflammatory response can also vary by condition, including network perturbation with resolution as in the case of pneumonia, early remodeling as in the case of asthma, degeneration and fibrosis as in the case of COPD, or proliferation as in the case of lung cancer. The extent of an individual's dysregulation at both the local and systems levels guides clinicians as to which interventions to undertake. In some cases, a patient's state may dictate the immediate use of suppressive or replacement therapy, complemented (or not) with bioregulatory therapy. In other cases the bioregulatory therapy might be the primary or even the only treatment approach.

**Figure 4:**
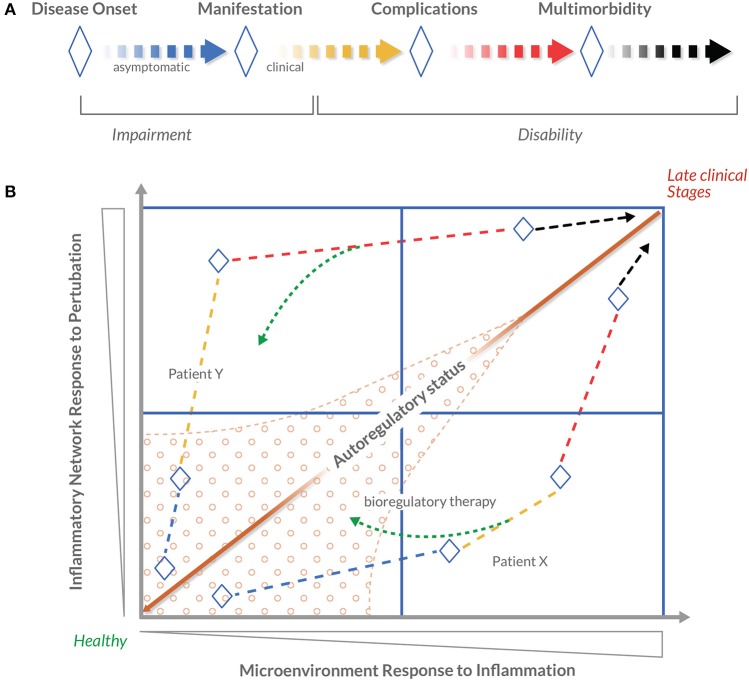
**Novel conceptualization of disease progression using patient autoregulatory status**. Disease progression is commonly understood as the worsening of a disease over time. In 1980, the World Health Organization published the International Classification of Impairments, Disabilities and Handicaps (ICIDH) with the objective of providing a widely accepted structure of the consequences of disease and the implications for the lives of patients. Expanding on this model, this figure presents a conceptualization of patient autoregulatory status. **(A)** The concept of disease progression, adapted from the 1980 WHO ICIDH model. Blue-to-black lines indicate stages in which a given disease is progressing, and ♢ depicts principal milestones between stages. The dashed line indicates that there is no strict sequential order between stages or milestones. A linear structure is used for simplicity. **(B)** A schematic conceptualization of disease progression as a four-quadrant map (using the BrSM model as a framework, disease progression is considered as the *Patient's Health-Disease Continuum*). The arrowed dashed lines represent the hypothetical disease progression of patients X and Y. In contrast to a more simplified, linear approach of identifying disease stages in clinical decision-making, this map positions stages in relation to dysregulation parameters represented by the horizontal and vertical axes. Within the context of the BrSM model, systemic dysregulation parameters are conceptualized as *Inflammatory Network Response to Perturbation* and local dysregulation parameters as *Microenvironment Response to Inflammation*. The farther along either axis a patient's disease progression is positioned, the greater the dysregulation. It is postulated that the ratio between these two dysregulation parameters theoretically defines the autoregulatory status of a patient. Mapping individual's autoregulatory status in a temporal fashion will produce a visualization of individualized disease progression. It is also postulated that a certain area of the map displays robust autoregulation capacity (marked area) in contrast to other areas where autoregulatory capacity is reduced. The therapeutic strategy proposed by the BrSM model proposes that a therapeutic effort is focused on “moving” a patient's autoregulatory status (represented by the green dashed arrows) to a state of more favorable autoregulation capacity. It is in this more favorable state where bioregulatory therapy can be most efficiently applied to strengthen autoregulation (hypothetical Patient X). It is assumed that in more advanced cases, it may not be possible to reach a state of favorable autoregulation capacity (hypothetical Patient Y). Bioregulatory intervention would be based on the patient's position on the map and, depending on the individual case, could serve as a primary, secondary, or complementary treatment to the suppressive or replacement therapy.

These clinical insights can be used to determine the degree of disease progression and, in turn, the appropriate clinical focus, therapeutic strategy, and medications with bioregulatory properties required to promote effective resolution (Figure 4). Depending on the condition and health history that a patient presents, the clinical focus and appropriate treatment will be individualized accordingly. Clinical focus may include the inflammatory network exclusively, the inflammatory and remodeling networks simultaneously, or also the metabolic network, blood sugar levels, or other physiological processes as well, depending on the patient. Likewise, appropriate treatment may include one or two medications with bioregulatory properties, or the use of bioregulatory treatment as an adjuvant to a patient's current treatment program. To date, this graded treatment approach is achieved through empirical assessment of the concepts in the clusters related to dysregulation (Figure 2). In the future, predictive biomarkers and diagnostics developed in the “omics” realm will provide a more in-depth, validated approach to assess the individual's autoregulatory capability in the presence of a stressor. Capturing dynamic changes would require multiple assessments during the treatment period. Therefore, an easily applied and inexpensive tool is desired. PCR-based biomarker panels can be purchased for as low as $20–40 per sample (Sahasrabudhe et al., 2014). This type of tool could take the form of a diagnostic index based on specific objective routine parameters, like FIB-4 or NIKEI in non-alcoholic fatty liver disease (Demir et al., 2013), or prognostically relevant clinical judgment questions (Ganna and Ingelsson, 2015), or as a blood-sample based biomarker panel (Mesko et al., 2010; Etheridge et al., 2011; Hu et al., 2014), or as a combination of all mentioned options. With next-generation sequencing costs falling rapidly, some solutions are already being tested for use in the clinical laboratory (Onsongo et al., 2014). Some studies indicate that next-generation sequencing solutions may become cost effective (Gallego et al., 2015; Li et al., 2015). Other recent evidence suggests that whole-genome transcriptomics captured at certain time periods before and during treatment could reflect the dynamics of transcriptome changes in response to perturbations and interventions (St. Laurent et al., 2013). Arguably, this would also allow for identification of any persistent perturbations that reflect dysfunctional autoregulatory cues. These trends indicate that potentially cost-effective biomarker-based tools are not outside of our reach. The two major challenges to BrSM are to reduce the costs of repeated diagnostic testing, and to identify effective responses for persistent perturbations.

Medication is an undeniably important part of the current healthcare approach and modern medicine more broadly. BrSM proposes an alternative conceptualization of medication—its design, how it is used—using lessons from the relevant scientific elements discussed prior. One objective of this conceptualization speaks specifically to the mounting evidence of side effects and other unintended consequences of many widely used medications developed under the current reductionist healthcare model. The reductionist or reactionary approach often uses medication under the guise of a “magic bullet” that can eliminate a disease cause or symptom. This single-target perspective tends to neglect consideration for how these medications unintentionally impact the overall regulatory ability of the human organism.

Consequently, model participants noted the potential for BrSM to include a novel approach to medication, rooted in the scientific principles that support the paradigm as a whole. Medications should be designed to mimic, modulate, or promote the body's natural resolution mechanisms instead of interfering with them. Of course, the precise design and application of such medications will depend on the specific context of a patient's condition, as described above. Nonetheless, the overall goal remains: Increase the ability to alter disease progression by promoting or mimicking resolution processes while incurring minimal side effects (Perretti and Dalli, 2009; Serhan, 2011; Rogerio et al., 2012). These concepts lead us to a key component of BrSM's therapeutic strategy: Interventions should utilize multitarget medications that act in concordance with multiple network interactions, feedback loops, and biorhythms inherent in autoregulatory networks. In other words, the efficacy of a medication in reversing the clinical picture of disease may be determined by its capacity to influence multiple interactions. As model participants endorsed, when multiple independent targets of the same pathways are inhibited simultaneously, a mild inhibition of each target can achieve a much larger therapeutic window and therapeutically relevant effect than single-target treatments (Csermely et al., 2005).

One important question is precisely how these medications can be derived. The notion of combination chemistry and synergy are viable strategies. Whereas the concept of synergy has been used for some time in various scientific disciplines, its use in medication design offers a novel application, and may be one that allows for more comprehensive and broader reaching effects. Combinatorial strategies may prove effective by inhibiting the pathophysiological pathways implicated in disease, while simultaneously altering other interconnected pathways that also influence disease regression. By employing a network pharmacology strategy, a medication can affect an entire signaling process. This strategy can include active agents that weakly target different proteins that are present in a given signaling network.

Put simply, a multicomponent strategy, applied through the lens of BrSM, has potential to influence a wide range of information flows in disease-perturbed networks, allowing for efficient control over such networks, and promising higher drug safety and less drug resistance. These benefits aim to trump the long-term effects of single component strategies, from which various undesirable consequences result. For BrSM, modest modulation, lower concentrations, and synergistic effects collectively suggest a potentially powerful adjuvant to the current medication model.

In sum, experts in this initiative clearly recognized an important link between multi-combination medication design and BrSM's scientific basis, noting that multitargeting, multicomponent medications can be used to purposefully influence biological information of regulatory networks and in turn impact reversal of disease. Perhaps the most beneficial aspect of this medication approach is in the potential to target multiple nodes of the autoregulatory networks involved in disease. This influence may not be limited to a particular target tissue or organ. As many disease-perturbed networks are present in many tissues, these medications may be able to address distorted information flow throughout a patient.

By addressing these underlying dysregulations through optimization of the autoregulatory system, the bioregulatory approach is potentially drug sparing and may lead to diminished incidence of iatrogenesis, patient morbidity, and patient mortality. We emphasize that above all, this approach is presented as a supplementary pharmacologic treatment that may lead to improved patient outcomes alongside other non-pharmacologic approaches. Although medicine holds important potential in changing health outcomes, we do not diminish the profound impact of other lifestyle behaviors such as diet and weight management that have proven to have a remarkable impact on chronic disease prevention (Ford et al., 2009).

## Discussion

At the most expansive level, participants summarized two primary undercurrents of BrSM: information regulation and disease resolution. We find these themes permeating all regions of the model, perhaps unsurprisingly given that the approach is purposed to bridge systems biology with a clinical application. Indeed, BrSM is advantageous in its proactive approach to disease management, supporting the temporal evolution of patient condition. In this regard, BrSM adds dynamic features to a historically static perspective of patient condition. This approach embraces change, both in terms of activity at micro and macro network levels, as well as in how disease resolution is achieved through endogenous and exogenous means.

The inflammatory response to network perturbation can be used to assess how well the autoregulation of physiological inflammation is able to induce homeostasis. The ability to observe non-resolution of acute physiological inflammation and a movement toward overwhelming acute inflammation (as seen in multi-trauma cases, for instance) or chronic inflammation, strongly suggests a breakdown in network information flow.

The microenvironment response to inflammation is another potential surrogate for assessing regulation capacity. As we can observe changes in the remodeling pattern of the microenvironment as a disease becomes increasingly chronic, it seems prudent to consider such remodeling as a natural indicator of disease progression. In accordance with this hypothesis, it is not surprising that markers of inflammation and matrix turnover are increasingly cited as predictive biomarkers of disease progression, e.g., in chronic stress (Hänsel et al., 2010), chronic prostatitis (Penna et al., 2007), atherosclerosis (Libby, 2002), chronic obstructive pulmonary disease (Vestbo et al., 2008), ankylosing spondylitis (Visvanathan et al., 2008), and non-alcoholic fatty liver disease (Wieckowska et al., 2006). Moreover, a novel inflammatory biomarker, YKL-40, is proposed as the clinically relevant alternative to CRP (Johansen, 2006).

Further research with “omics” platforms, especially genomics, will likely validate, refine, or even replace the use of these surrogates in clinical decision-making. These platforms are especially well-suited for a systems medicine approach, as they embrace the analytic complexity of biological networks, and are therefore also likely to assist in identifying the various targets of a multicomponent medication design.

### Areas for future research

Like many recent advances in medical theory development, BrSM faces various challenges in actualizing its clinical potential. Participants acknowledged that much ongoing research is needed to validate and expand its scientific and clinical evidence base. In this section, we summarize the major areas for future research concentration that will support the practical application of the BrSM approach. Our hope is that this paper encourages scientific and clinical communities to explore these areas in their own work, and consequently help to propagate the growing need for more effective therapeutic strategies.

The ability to measure the multiple networks involved in disease processes is a critical step in addressing disease at the systems level and understanding the global autoregulatory network. Whole genome transcriptome analysis provides an optimal analytic tool for understanding the genomic quantification of disease evolution and health-disease status. High resolution transcriptome maps of disease will allow for the identification of therapeutic targets and will further guide diagnosis and medication design, thereby enhancing the practical value of the BrSM model. The advent of next-generation sequencing methods will also allow for a more individualized picture of health and disease, further advancing personalized medicine (Sripada et al., 2012).

Research on the microenvironment or “terrain” in which inflammation takes place will provide more comprehensive insight into treating the underlying causes of chronic inflammatory conditions, extending the therapeutic value of any medical intervention beyond targeting symptoms alone.

Chronic inflammation, however, is biologically complex; therefore, the same intervention could produce different effects in different patients at different times (Nathan and Ding, 2010). Future therapeutic systems would benefit from the ability to assess the current inflammatory profile of individual patients, which could then help to identify and locate any blocks to resolution, as well as underlying pathologies.

Furthermore, the ability to measure the history and culmination of an individual's resolution factors over time can allow the clinician to better evaluate the overall inflammatory status of a patient and prescribe the most appropriate treatment.

It is important to note that the inflammatory network is not the only perturbed system of a particular disease, or even the main target of the therapeutic approach. The endocrine, neurological, and other systems are affected as well. We posit that the clinical picture of inflammation may nonetheless be used as a surrogate marker to classify known diseases in order to predict the status of the autoregulatory network.

Development of a formal diagnostic platform for BrSM is likely to aid in realizing and validating the relationships among its scientific and clinical elements. Diagnostics are also essential in making therapeutic decisions. The ability to evaluate the autoregulatory patterns of a patient is critical in determining the appropriate combination of treatments to achieve homeostasis. Although empirical inflammatory patterns or allostatic state models may provide useful surrogates for measurement in the absence of formal diagnostics (Romero et al., 2009; Oken et al., 2014), genomic and other “omics” patterns are likely to better delineate the autoregulatory status of a patient. The information elicited from genomic patterns can potentially address the need to better understand the scientific basis that relates the more conceptually sound anchors of the model.

Appropriate diagnostic technological platforms are also essential for capturing relevant biological information at the necessary level of detail. In the context of BrSM, lipidomics, metabolomics, genomics, and proteomics are technologies that can help to detect and monitor the autoregulatory state of a patient in order to diagnose more comprehensively. New technologies will also allow for numerous markers to be tested simultaneously, expanding the diagnostic utility of already commonly tested clinical fluids such as blood (Hood and Flores, 2012), saliva (Zauber et al., 2012), and urine (Sharma et al., 2011). In the near term, “omics” technologies are relatively expensive because they are still evolving and are quantifying the entire genome, transcriptome, or proteome. In 3–5 years, as these technologies become more price competitive and the relevant genes, transcripts, and proteins become well known, focused “omics” tests will be fast and inexpensive methods for guiding BrSM therapy.

Multitargeting medications suggest a promising pathway for influencing biological information of regulatory networks, and with a greater degree of agency and purpose than current widely used medications (Csermely et al., 2005). Given the current healthcare and medical challenges, it is evident that the single-molecule, single-target paradigm does not provide the specificity and sophistication that a multitargeting model has potential to offer. To this end, new medications and treatment protocols are warranted to target and bioregulate perturbed autoregulatory networks toward resolution. In addition to new medication design, this strategy can be applied to the vast dataset (Comprehensive Medicinal Chemistry^2^) of existing drugs to create new, unique formulas. This approach is already used for cancer therapeutics, which currently include eight drugs that inhibit more than one regulatory enzyme. Evidence shows that this multiple target activity is advantageous in an oncology setting (Knight et al., 2010).

A multitargeting medication strategy inevitably raises questions about the number of known molecular targets that can be used for future combination design. Whereas current databases include targets derived from biological information sequencing, there is little evidence of bioregulatory network information. Future database development that considers the complexity of regulatory information will likely expand the drug target landscape to unprecedented levels. Nonetheless, existing knowledge can support the design of medications that will act on multiple targets across known disease networks.

At this point, three drug design strategies can be suggested. First, multiple individual medications can be used simultaneously. This strategy is most in line with current medical approaches, as in the case of treatment protocols for HIV, tuberculosis, primary hypertension, osteoarthritis, lupus erythematosus, metabolic syndrome, fibromyalgia, and others. Second, multicomponent medications can be developed that contain two or more active ingredients (e.g., Combivir, Atripla, Advair, Caduet, Iberogast, Traumeel). Third, single-component medications can be developed that act on multiple targets simultaneously. This is the major objective of chemogenomics, a novel pharmacology field (Medina-Franco et al., 2013; Sakharkar et al., 2015). BrSM embraces all three strategies, to the extent that they support the intent of neither blocking nor interfering with endogenous resolution pathways that help to reduce therapy side effects and promote long-term benefits.

Finally, the potential to influence stem-cell niches through medication begs the question as to whether using niches as drug-targets may be a valuable treatment component. Stem cells exist in niches which act as basic physiological units that integrate signals in order to mediate stem cell response to organism needs. Niches essentially regulate the extent to which stem cells are involved in tissue repair, generation, and maintenance. While niche manipulation has been broadly considered in the context of various chronic conditions (e.g., cardiac repair, diabetes, cancer) (Department of Health and Human Services, 2006), these concepts may also apply to regulation and mediation of chronic inflammatory conditions. By targeting the inflammatory system in treatment, stem cell niches may be influenced and in turn impact the regeneration of affected tissue.

Future clinical research may consider whether bioaccumulation of toxins in tissues can negatively influence cellular health, as environmental toxins and metabolic waste products can accumulate in the extracellular matrix and cause disease. Recent research in various health disciplines demonstrates that deficiency and toxicity are common etiological determinants of contemporary ill-health (Genuis, 2012). Bioaccumulation of pesticides in adipose tissue, for example, increases the total body burden of intoxication and may lead to neural, immune, and endocrine toxicity (Crinnion, 2000). This line of research also suggests a relationship between bioaccumulation of environmental toxins and perturbations in the immune response/inflammatory network that should be explored further in the context of BrSM.

The ability to identify molecular networks shared by the progression of multiple diseases suggests that modeling an individual's health-disease continuum would also provide clinically relevant information. In one study, whole-genome sequencing was applied to a blood sample from a patient with a history of vascular disease and early sudden death as a means of developing a model of the patient's individual disease network. The resultant model displayed an interconnected picture of disease modifiable factors such as smoking, diet, alcohol, exercise, and medication use, as well as risks for developing coronary artery disease, obesity, osteoarthritis, and Type 2 diabetes. Given the high correlation among these diseases, the authors concluded that information regarding individual patient disease risk and response to drugs can in fact be derived from whole-genome sequence data (Ashley et al., 2010). In another example, researchers identified a set of molecular networks that are perturbed during the progression of a prion disease in mouse models. The researchers monitored the global molecular information from onset, to the appearance of symptoms, to the final disease stages. Interestingly, the four identified networks of the prion disease are also those perturbed in other neurodegenerative diseases such as Alzheimer's disease, Huntington's disease, Parkinson's disease, and amyotrophic lateral sclerosis. This line of research further supports the potential insight derived from understanding and identifying molecular networks that are shared by the progression of multiple diseases, and applying this insight to modeling an individual's disease evolution (Hwang et al., 2009).

From a clinical practice perspective, this research suggests that in any individual patient, disease interconnectedness (by shared molecular events) represents the individual's disease evolution, reflected in the patient's medical history. The results support the development of personalized medicine, and provide an opportunity for the disease evolution concept to be formally introduced and studied in the clinical setting. Some research groups within the scientific medical community have already begun developing disease models, such as in the case of diabetes, and have validated these models in an RCT setting (Eddy and Schlessinger, 2003). The success of this modeling supports future research that looks at simulating the dynamic evolution of health-to-disease processes in a way that can be used to predict the response of a whole inflammatory/wound-healing biological network, rather than the response of particular inflammatory mediators.

## Conclusion

Although various challenges remain in bringing BrSM to patients, the foundation outlined in this model offers fruitful grounds for next steps. Looking ahead, the BrSM scientific community should pursue focused research projects that aim to establish the molecular fingerprint of autoregulatory networks, map the health-disease continuum, improve autoregulatory capacity diagnostics from empirical to objective assessment tools, and test various bioregulatory treatment strategies in clinical settings. Pharmacoeconomic studies evaluating cost-effectiveness of BrSM diagnostics and therapies will become an important part of this research. Ongoing research in the fields of systems biology also promises to strengthen the scientific landscape of the BrSM approach. Empirical evidence from clinical experience and the development of patient registries will continue to validate its ability to resolve chronic conditions.

The interconnectivity that sustains the human organism is undeniable. We anticipate that approaches like BrSM can provide roadmaps for connecting the dots between scientific discoveries and ideal clinical outcomes. While we are still in the early stages of this paradigm shift, emerging conceptual models such as that presented in this paper promise to pave the way for a future of medicine that is cost effective, patient-centered, and better able to achieve improved medical results.

### Conflict of interest statement

Biologische Heilmittel Heel GmbH has financially supported the organization of International Think Tanks; Concept Systems, Inc has received financial support from Biologische Heilmittel Heel GmbH for project management; David Lescheid, Martha Herbert, Timothy McCaffrey, Georges St. Laurent III and Brian Berman have received honorariums from Biologische Heilmittel Heel GmbH; Georges St. Laurent III has received research grants from Biologische Heilmittel Heel GmbH. Alyssa W. Goldman and Mary Kane are employees of Concept Systems, Inc. Yvonne Burmeister, Konstantin Cesnulevicius, Myron Schultz, Bernd Seilheimer, Alta Smit are employees of Biologische Heilmittel Heel GmbH.

## Footnotes

^1^CASYM. Europe - Coordinating Action Systems Medicine. Implementation of Systems Medicine across Europe. Available online at: www.casym.eu

^2^Comprehensive Medicinal Chemistry (CMC). Available online at: http://accelrys.com/products/databases/bioactivity/comprehensive-medicinal-chemistry.html
