# Supplementary material for: Bioregulatory systems medicine: an innovative approach to integrating the science of molecular networks, inflammation, and systems biology with the patient's autoregulatory capacity?
Source: Front Physiol. 2015 Aug 19;6:225. doi: 10.3389/fphys.2015.00225 (PMC4541032; doi:10.3389/fphys.2015.00225)
Supplement: Supplementary file 1 [file Table1.DOC]

| **#** | **Cluster 1: Microenvironment Response to Inflammation** |
| --- | --- |
| 1 | The extracellular matrix is involved in the initiation and resolution of the inflammatory response. |
| 5 | An active lymphatic system that promotes lymphatic drainage and cell migration is essential for the resolution of inflammation. |
| 25 | The detection of inflammatory mediator patient profiles could help to identify and locate resolution blockages and underlying pathologies. |
| 42 | The communication between the cell and microenvironment is bidirectional, and forms the basis of the homeostatic control of many tissues. |
| 47 | The treatment of a wide range of human disorders could be improved by stimulation or optimization of the patient's individual inflammation resolution process. |
| 91 | The global autoregulatory network, including input from neural and hormonal pathways, influences the overall form and function of the extracellular matrix. |
| 98 | Inflammatory reactions often occur within distinct microenvironments composed of tissue-specific cells, (fibroblasts, endothelial cells, and macrophages) and their highly specialized extracellular matrix (ECM) components. |
| **#** | **Cluster 2: Biological Communication at the Microenvironment-Scale** |
| 39 | The extracellular matrix is the part of the immunological synapse that occurs in the cell microenvironment between antigens and antigen presenting cells. |
| 62 | The extracellular matrix, intracellular cytoskeleton and nuclear matrix are directly interconnected through a chain of commonly utilized molecules. |
| 66 | The extracellular matrix is involved in the progression of almost any chronic disease, most prominently in any fibrotic disease, most solid tumors, arthritis, osteoporosis, COPD and emphysema. |
| 67 | Excessive breakdown of the extracellular matrix components is associated with altered levels of reactive oxygen species (ROS) that can result in modification of multiple molecular networks and subsequent pathology. |
| 68 | The extracellular matrix links signals from the microenvironment (e.g. neural, hormonal, biochemical and biophysical) to the cytoplasm and then the nucleus, thereby directly influencing transcription patterns. |
| 79 | Environmental toxins and metabolic waste products can potentially accumulate in the extracellular matrix and cause disease. |
| **#** | **Cluster 3: Inflammation Physiology** |
| 2 | Local inflammatory pathways and mechanisms in the body are mirrored by and related to systemic inflammation, which is one of the underlying pathological mechanisms of many diseases. |
| 6 | Inflammation where the mechanisms of normal resolution are inadequate or suppressed as well as persistent low grade inflammation resulting from an inability to mount an adequate inflammatory response are the major causes of many diseases. |
| 15 | Chronic inflammation often leads to tissue injury, scarring and fibrosis. |
| 17 | Inflammation is a part of the immune response, which can be triggered by exogenous and endogenous stimuli in either non-sterile or sterile environments. |
| 36 | Acute inflammation supports the removal of damaged tissue. |
| 41 | The balance of pro- and anti-inflammatory factors, including external signals, determines the inflammatory status. |
| 43 | The purpose of any acute inflammatory response is to eliminate disturbances that are interfering with normal conditions and therefore restore functionality and homeodynamics/homeostasis to the tissue. |
| 50 | The physiological mechanisms of inflammation are necessary to maintain health and to return from the disease state to a homeostatic healthy state. |
| 52 | Resolution of inflammation is steered by multiple endogenous anti-inflammatory and proresolution molecules and pathways. |
| 74 | Inflammation that is suppressed during its normal pathway or is non-resolving causes or contributes to pathological states. |
| 83 | Inflammation is regulated by an orchestra of molecules, and persists whenever a component of the complex signaling pathway fails or gets lost. |
| 84 | An acute inflammatory response is an adaptive response that should not be blocked and possibly even initiated in order to induce resolution and restore homeodynamics/homeostasis in a tissue. |
| 88 | The ideal outcome of acute inflammation is complete resolution. |
| 94 | There are many different potential triggers for inflammation other than damage and infection including dysregulated cell metabolism, hyperpermeable mucosal membrane barriers and alterations of the extracellular matrix. |
| 95 | Certain physiological functions (e.g. epithelial cell turnover in the intestinal tract for the maintenance of barrier integrity) rely on a constitutive level of inflammatory signals. |
| **#** | **Cluster 4: Inflammatory Network Response to Perturbation** |
| 4 | Inflammation has various physiological purposes and is induced by exogenous as well as endogenous stressors released during tissue injury, tissue stress and malfunctioning. |
| 19 | The loss of molecular order in the cell triggers inflammation. |
| 21 | Functionally capable auto-regulating tissue induces acute inflammation response when molecular order is lost in an effort to restore and maintain order in the system. |
| 22 | The human body has the capability to synthesize and control regulatory molecules that promote and resolve inflammation. |
| **#** | **Cluster 5: Clinical Focus on Dysregulation** |
| 3 | Atopic diseases, as examples of Th2 regulation rigidity, are well suited for bioregulatory intervention due to the fact that only symptomatic medical solutions are currently available. |
| 9 | Bioregulatory systems medicine supports the body's autoregulatory system, thereby triggering endogenous inflammatory mediators and mechanisms that optimize the (time) course of inflammation resolution. |
| 14 | Evaluating a disease within disease-health continuum is an important tool for bioregulatory systems medicine in order to design the treatment and follow-up. |
| 16 | When certain heritable diseases and diseases where organ failure and tissue damage are at the point at which auto-regulation is impossible to restore, bioregulatory systems medicine can be used to treat symptoms and prevent sequelae, rather than as a standalone treatment. |
| 26 | The variety of pathways and molecules involved in the complex response to inflammation points to the necessity of multitargeting/multicomponent medications. |
| 46 | Functional somatic syndromes including rhinitis, fibromyalgia and chronic fatigue syndrome often share similar blocks to autoregulation. |
| 57 | Chronic diseases associated with aging can be potentially better managed with a multitargeting approach. |
| 59 | A patient's individual position along the health-disease continuum in conjunction with the identified stressor(s) will determine the treatment strategy. |
| 63 | Bioregulatory systems medicine can serve as an adjuvant treatment to reduce polypharmacy, provide effective and safe relief of symptoms, and prevent cascade iatrogenesis. |
| 64 | Functional somatic syndromes are excellent targets for bioregulatory systems medicine due to their multifactorial network pathophysiology and the lack of effective medical solutions currently available. |
| 69 | Numerous diseases including asthma, chronic rhinosinusitis, atopic eczema, chronic fatigue syndrome and fibromyalgia syndrome are all influenced by a breach in the integrity of epithelial membranes. |
| 75 | When treating conditions of severe regulation rigidity without adequate and timely restoration of regulation, a more comprehensive treatment program is necessary to remove all stressors and blocks to auto-regulation/compensation, and to apply the appropriate courses of bioregulating medicines. |
| 96 | Chronic disease management should move towards holistic, multi-modal integrated care, and multi-scale, multi-level system approaches. |
| **#** | **Cluster 6: Bioregulatory Clinical Pharmacology** |
| 7 | Multi-combination and/or multi-system low dose medications, preferably of natural origin, are well suited for the bioregulatory medical approach and offer the potential for a graded response to treatment. |
| 12 | Inhibitory pharmacologic intervention is an option of choice when a single disease causative factor is identified and must be eliminated and there is insufficient time to complete a proper bioregulatory treatment (e.g. the treatment of acute MI or stroke). |
| 31 | Bioregulatory systems medicine is a method of choice in treating multifactorial disease when restoration of homeodynamics is still achievable. |
| 34 | A multicomponent, multitargeting medical management model may be a solution to current inadequate treatments for multi-factorial diseases such as dementia, certain cancers, cardiovascular disease and metabolic disorders such as Type II Diabetes and metabolic syndrome. |
| 45 | The functions of medications with bioregulatory properties are determined by natural combination chemistry and synergy. |
| 51 | The efficacy of a complex medication is determined by its ability to influence multiple interactions to reverse the clinical picture of disease. |
| 53 | Multicomponent medications target multiple nodes of a perturbed molecular network simultaneously. |
| 54 | Bioregulatory therapies should be considered in the context of biological rhythms. |
| 71 | Medications with bioregulatory properties can act on multiple organ systems and multiple targets in disease-related molecular networks simultaneously. |
| 72 | Biological information of regulatory networks can be directly and purposefully influenced with multitargeting and multicomponent medications. |
| 77 | When multiple independent targets of the same pathway are inhibited simultaneously, a mild inhibition of each target is sufficient to achieve a much larger therapeutic window and a therapeutically relevant effect. |
| 90 | Medications that neither block nor interfere with endogenous resolution pathways will help to reduce therapy side effects and promote long-term benefits. |
| 92 | Medications with bioregulatory properties influence tissues by helping to restore molecular coherence. |
| 99 | The concurrent and gentle use of more than one natural substance in alignment with a network medicine approach may offer a safe and effective alternative to the current medical paradigm. |
| **#** | **Cluster 7: Diagnostics and Therapeutic Strategy** |
| 10 | Fully integrated bioinformatical models will help to mechanistically explain disease states and support the development of targeted therapeutic strategies. |
| 28 | Treatment of a symptom alone, without considering the underlying cause, can disturb the autoregulatory process. |
| 29 | A more detailed molecular picture of disease evolution will lead to novel treatments, which may involve targeting whole networks. |
| 30 | Bioregulatory medical interventions can range from supporting auto-regulatory capacity to actively provoking a stimulus to restore and clear the blocks to auto-regulation capabilities. |
| 35 | Integration of all molecular diagnostic techniques will provide a more detailed picture of disease evolution. |
| 60 | Novel diagnostic solutions, including measuring heart rate variability, complex molecular biomarker panels and omics technologies including whole-blood deep sequencing, will allow for the assessment of the global auto-regulation/compensation state and the organism’s response to the bioregulatory treatment. |
| 61 | Therapeutic decisions in bioregulatory systems medicine are made based on the capacity of the affected autoregulatory network in relation to the causative stressor. |
| 73 | In diseases with a chronic relapsing course and relatively good health during the remission period, regulation can be regained by eliminating the stressor (spontaneously or via appropriate medical intervention), clearing the block to auto-regulation, or supporting the auto-regulatory network. |
| 76 | The modeling of a disease as a molecular/cellular network will lead to the development of novel diagnostic test systems tailored to multitargeting therapies. |
| 80 | The degree of the body's dysregulation can be classified into basic patterns which then serve to make therapeutic decisions. |
| 86 | Medications with bioregulatory properties should not permanently interfere with the body's auto-regulation networks. |
| 93 | A clinical model that guides therapeutic decision-making based on assessment of tissue molecular networks in the context of the patient's auto-regulatory ability is better suited for accurate prediction of disease outcomes, intervention follow-up and disease prevention. |
| 100 | Diagnostic measurements should be expanded beyond current markers to include the assessment of autoregulatory networks and blocks to autoregulation. |
|  | **Cluster 8: Patient Health-Disease Continuum** |
| 37 | Symptoms are an expression of the response of the autoregulatory system to a stressor. |
| 58 | The progression of a disease is facilitated by disturbed or inadequate autoregulatory abilities of the organism. |
| 65 | In any individual patient, disease interconnectedness (by shared molecular events) represents the individual's disease evolution, reflected in the patient's medical history. |
| 78 | Simulating the dynamic evolution of health-to-disease processes can be used to predict the response of a whole inflammatory/wound-healing system, rather than the response of particular inflammatory mediators. |
| 97 | Disease progression is the result of an auto-regulatory process that is disturbed or challenged by an overwhelming stressor and cannot function adequately to restore homeodynamics. |
| 102 | Lipidomics, metabolomics, genomics and proteomics are technologies which can help to detect and monitor the inflammatory state of a patient in order to diagnose more comprehensively. |
| **#** | **Cluster 9: Autoregulation of Biological Networks** |
| 8 | A multi-scale network of all molecular components and their within- and cross-tissue interactions can serve as a global autoregulation model of the human organism. |
| 20 | Blocks to autoregulation are etiological factors that maintain persistent network perturbation and restrict the network from autoregulating towards resolution. |
| 23 | There is a high level of molecular coherence in healthy tissues and the loss of molecular order corrupts "healthy" information flow in the tissue. |
| 27 | Robust molecular networks are able to autoregulate in order to restore or adapt its functional state in response to external inputs. |
| 48 | Disease progression is characterized by an increase in the thermal degrees of freedom and, as a result, a decrease in the molecular coherence of the affected tissues. |
| 49 | Sustained corruption of "healthy" information flow leads to the failure of regulatory networks' ability to restore molecular order. |
| 55 | Diseases that share molecular/cellular networks show phenotypic similarity and comorbidity (e.g. the link between atherosclerosis and obesity). |
| 56 | The majority of diseases share a certain number of common molecular functional modules, each associated with a pathophysiological process. |
| 70 | A network approach can be used to identify common pathological threads between seemingly unrelated diseases, to improve the understanding of the pathogenesis and therefore, to aide in the discovery of the most influential therapeutic access points. |
| 81 | Signals from the microenvironment directly influence many functional modules of molecular networks representing physiological processes, including angiogenesis, development of certain glands and wound healing. |
| 87 | Robustness is the ability to maintain homeodynamics/homeostasis of living systems in the face of perturbations and uncertainty. |
| 89 | Persistent perturbation of molecular networks, including endogenous responses to specific exogenous insults, manifests as disease. |
| 101 | Many diseases are interconnected by shared molecular events. |
| **#** | **Cluster 10: Biological Communication across Multi-Scale Networks** |
| 11 | The informational nature of a human organism as a biological system allows for the creation of mathematical models of health and disease. |
| 13 | A science of systems biology is a holistic approach in biology focused on understanding complex interactions in biological systems. |
| 18 | There are two major types of biological information: sequence information encoding molecular machines and regulatory network information controlling the behavior of the molecular machines. |
| 24 | Biological networks are inherently unstable and dynamic; their capability to adapt against constantly changing internal and external inputs is dictated by their robustness. |
| 32 | Information in the living system can be digital (e.g. 4-digits nucleotide code) or analogous (e.g. 2D-3D spatial structures of molecules). |
| 33 | Tissues and organs can be linked together in networks by the functional interdependencies between them. |
| 38 | Information theory and thermodynamics are fundamental for understanding the principles of a biological system. |
| 40 | Molecular coherence, which can be defined as the behavior of molecules in the tissue in response to the whole network of all other molecules can be quantified as the ratio between codable systems and thermal degrees of freedom. |
| 44 | Much of the complexity of living organisms stems from complex regulatory networks, rather than from gene diversity. |
| 82 | Low affinity interactions (especially RNA-protein interactions) provide a computational matrix to process information and to direct action in molecular networks. |
| 85 | Molecules are informational units that circulate in non-linear, network mode. |
